# Are There Benefits to Breastfeeding for Long Durations That Continue after Breastfeeding Has Stopped? An Analysis of Acute Respiratory Illness in Nigerian Children †

**DOI:** 10.3390/children11091144

**Published:** 2024-09-21

**Authors:** Lilian Ouja Ademu, Rajib Paul, Elizabeth F. Racine

**Affiliations:** 1 Texas A&M AgriLife Research Center at El Paso, El Paso, TX 79927, USA; 2 Department of Epidemiology and Community Health, College of Health and Human Services, University of North Carolina at Charlotte, Charlotte, NC 28223, USA; rpaul9@charlotte.edu; 3 Department of Nutrition, College of Agriculture and Life Sciences, Texas A&M University, College Station, TX 77843, USA; beth.racine@ag.tamu.edu

**Keywords:** breastfeeding, breastfeeding duration, acute health outcomes, sub-Saharan Africa, Nigeria

## Abstract

**Background:** While an abundance of evidence exists regarding infectious outcomes in children as they relate to the short-term benefits of breastfeeding, there is limited evidence related to similar impacts beyond one year and after breastfeeding has stopped. Specifically, little is known about the long-term benefits of breastfeeding for acute health outcomes after infancy, particularly in Nigeria. **Methods:** The Nigeria Demographic and Health Survey data was used in this study. We utilized data (n = 5391) on children who had stopped breastfeeding for at least 12 months before the survey. Breastfeeding duration was categorized into 1–6 months, 7–12 months, 13–18 months, 18–24 months, and > 24 months. Any recent incident of acute respiratory illness in children was operationalized using the responses to related questions (recent incidents of fever, cough, running nose, and short, rapid, or difficulty breathing in children). Adjusted logistic regression was used to estimate odds ratios, and statistical significance was determined at *p* ≤ 0.05. **Results:** Post-infancy and after breastfeeding had stopped, the odds of recent acute respiratory illness were significantly less (AOR = 0.37, 95% CI [0.15–0.79], *p* = 0.04) in children breastfed for 19–24 months compared to those breastfed for 1–6 months. No significant association was found between the other durations and ARI post-infancy (*p* > 0.05). **Conclusions:** These findings indicate that breastfeeding for up to 24 months has a long-term protective effect from an acute health condition that contributes to the high under-five mortality rates recorded for decades in Nigeria specifically, and more broadly, in sub-Saharan Africa.

## 1. Introduction

A World Health Organization (WHO) recommendation for optimal Infant and Young Child Feeding (IYCF) is that after 6 months of exclusive breastfeeding, complementary breastfeeding continues until a child is 24 months [1]. Well known for its rich content of nutrients, antimicrobial, anti-inflammatory, and immune factors, breast milk is a uniquely suited infant nutrition and health intervention, especially in countries lacking extensive health and nutrition support [2,3].The macronutrient composition of human milk varies both within individual mothers and throughout the stages of lactation. Despite these variations, human milk, especially colostrum, is notably rich in immunological components such as secretory IgA, lactoferrin, and leukocytes, as well as developmental factors like epidermal growth factor [4]. The health benefits of breastfeeding for infants and children have been extensively investigated [5,6,7,8,9]. Yet few studies have examined the long-term benefits of breastfeeding in reducing incidents of acute illnesses in under-five children after infancy [10,11,12,13,14], particularly in sub-Saharan Africa (SSA), a region that has contributed to the global under-five mortality rates for many decades [15,16].

Acute illnesses remain the leading cause of child morbidity and mortality in under-five-aged children in SSA, and children less than five years old in the region are 14 times more likely to die from diseases compared to their counterparts in developed regions [16,17]. Nigeria currently accounts for one of the highest morbidities and deaths in children under five years of age in the region [15,16] and Acute Respiratory Illness (ARI) is one of the major contributors to this burden [18]. In 2022, the national under-five mortality rate in Nigeria was 107 per 1000 live births [19]. One of the Sustainable Development Goals (SDGs), established by the United Nations General Assembly in 2015, aims for all countries to achieve an under-5 mortality rate of no more than 25 deaths per 1000 live births and a neonatal mortality rate of no more than 12 deaths per 1000 live births by 2030 [15].

The short-term benefits of breastfeeding are the immediate benefits a child enjoys while they are being breastfed, while the long-term benefits are the associated benefits after breastfeeding has stopped [12]. While the short-term effects of breastfeeding on acute illnesses in childhood have been extensively studied [20,21,22,23], comparatively fewer studies have explored its long-term benefits, especially for acute illnesses in children post-infancy [24,25]. Many of the studies examining its long-term effects have focused on chronic and non-communicable diseases like obesity, diabetes, cancer, and cardiovascular diseases in later years [13,26,27,28,29]. Except for a few studies [12,24,25], research on the long-term benefits of breastfeeding on acute illnesses in early childhood is relatively scarce, especially in SSA. To the best of our knowledge, no research has explored the long-term effects of different breastfeeding durations on Acute Respiratory Illness (ARI) outcomes after infancy in Nigeria. 

Nigeria is the most populous country in SSA. With a population of over 200 million, it has contributed to the high mortality rates reported among children living in SSA for many decades [15,30]. While the overall burden of childhood diseases has reduced in Nigeria over the years, childhood morbidity is still a major challenge in the country [18]. 

This study uses data from the Nigerian Demographic and Health Survey (NDHS) to examine if there are long-term benefits to breastfeeding for longer durations. It specifically examines the impact of different durations of breastfeeding on ARI after breastfeeding has stopped; in children aged 24 to 59 months. We hypothesize that children who were breastfed for longer than 6 months would have fewer recent incidents of the disease long after breastfeeding has stopped.

## 2. Materials and Methods

### 2.1. NDHS Study Design and Data

The Demographic and Health Surveys are nationally representative surveys conducted in many low- and middle-income countries in collaboration with the United States Agency for International Development. They are also considered to be the principal sources of population health data in developing countries, especially where comprehensive health data are otherwise absent [31]. This cross-sectional study uses the 2008 NDHS data, which contains information on breastfeeding practices, breastfeeding duration, and health indicators for all children surveyed.

The NDHS survey provides cross-sectional data on the demographics, socioeconomic characteristics, nutrition, and health indicator estimates of the population in Nigeria. Data are obtained from randomly selected households across the six geopolitical zones in the country. The 2008 NDHS applied a stratified two-stage cluster design to select study participants from a complete listing of households and a mapping exercise carried out for each cluster from April to May 2008. More information on the sampling design, sampling technique, and sample size determination is available in the 2008 NDHS country report [32]. 

The 2008 NDHS dataset are the most recent survey data with information on breastfeeding duration for every child below 3–5 years in each household included in the survey. After 2008 and for surveys based on the DHS-VI, DHS-VII, or DHS VIII, information on the duration of breastfeeding, antenatal, and acute health outcomes is only collected for the youngest children or those who were still breastfeeding at the time of the survey [33]. This is why this survey was used for this study. 

The women’s questionnaire of the survey collects data on breastfeeding, maternal and child health, and recent incidents of acute health outcomes in children. Information on breastfeeding duration, childbirth size, antenatal visits, mode of delivery, recent fever incidents, diarrhea, cough, running nose, and short and rapid breaths in children is based on the mother’s recall. 

### 2.2. Sample

**Figure 1:**
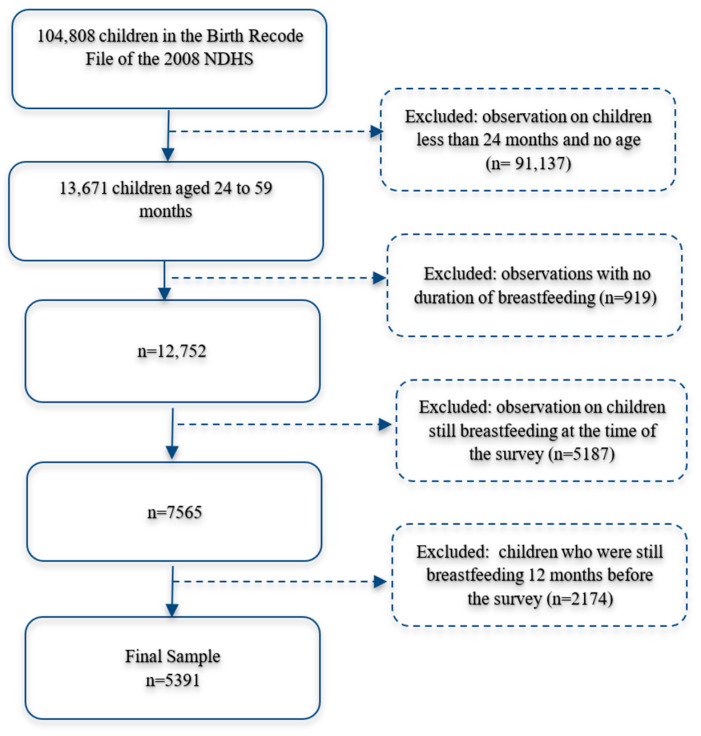
Inclusion and exclusion criteria for the sample selection.

Figure 1 shows the inclusion and exclusion criteria used in the sample selection. Only breastfed children aged 24 to 59 months and who had stopped breastfeeding for more than 12 months were included in the final sample (n = 5391). The DHS measures “when child put to breast” to measure early initiation of breastfeeding for mothers who breastfed [34]. We assume that most of the children in the sample who were breastfed received breastmilk within the first month of the child’s life since all the children with available data in the sample were breastfed within 2 days. Thus, a child’s age minus the duration of breastfeeding was used to estimate the months since breastfeeding stopped.

### 2.3. Outcome Variable 

The outcome variable in this study is any recent incident or episode of ARI in the two weeks preceding the survey. This outcome was operationalized from yes or no (0/1) answers to the following questions asked in the survey questionnaire: (a) Has the child been ill with a fever in the last 2 weeks? (b) Has the child had an illness with a cough in the last 2 weeks? (c) When the child has an illness with a cough, did he/she breathe faster than usual with short, rapid breaths or have difficulty breathing in the last 2 weeks? A recent incident of ARI in a child was recorded and coded as one if a child’s mother’s response to all of the questions (a), (b), and (c) was “yes” [32,35].

### 2.4. Exposure Variable 

The main exposure variable in this study is the duration of “any breastfeeding”. This is typically a practice in which a child is either exclusively, predominantly, partially, or fully breastfed. In other words, if a child receives breastmilk with or without complementary foods or liquids [1,36,37]. Following the methodology employed in past studies, this variable was grouped into five categories: 1–6 months, 7–12 months, 13–18 months, 18–24 months, and >24 months [38,39]. 

### 2.5. Covariates

Factors such as maternal education, household wealth, source of cooking fuel, place of residence, nutrition, and other maternal, demographic, and household environmental factors have been associated with childhood morbidity [40,41,42,43,44,45]. Covariates adjusted for in the analysis are the mother’s age (in years), the child’s age (in months), the sex of the child (male or female), the mother’s educational level [46], mother’s employment status (in the past 12 months), the mother’s marital status [12,24], number of antenatal visits during pregnancy, mode of child delivery (caesarean or natural birth), place of residence (urban or rural), the wealth index of the household, the use of a mosquito bed net by a child, the household’s type of cooking fuel, the nutritional status of the child (weight-for-age, height-for-age, and weight-for-height), birth size of a child, and the geographic location of a household [18]. 

The DHS data “wealth index” variable is classified into quintiles using principal component analysis. This index is based on household ownership of consumer goods (e.g., television, bicycle, car) and housing characteristics (e.g., source of drinking water, toilet facilities, flooring materials) [47]. This variable was used as recorded in the survey. Also, the DHS reports indicators for the nutritional status of children using height-for-age (HAZ), weight-for-age (WAZ), and weight-for-height (WHZ) as standard deviations. For our analysis, the z-scores were used as recorded in the 2008 NDHS and categorized as vectors capturing different degrees of stunting, underweight, and wasting in children [48,49]. For example, children who were below −2 standard deviations (SDs) from the median of the WHO Child Growth Standards in weight for age were categorized as underweight [32]. Similarly, children who were below −2 SDs for height for age (HAZ) and weight for height (WHZ) were categorized as stunted and wasted, respectively [48]. Lastly, birth size in the DHS is measured through maternal recall of five categorical descriptions of a child’s size: very small, smaller than average, average, larger than average, or very large. We used this categorization in our analysis. More details on the categorization and coding of all the covariates are provided in the Supplementary File (Table S1). 

### 2.6. Statistical Analysis

A multivariate logistic regression model was used to examine the long-term effect of the five categories of breastfeeding duration on recent incidents of ARI in children aged 24 to 59 months [35]. We estimate adjusted odds ratios using the model described below:

*logit* (*Pr* (*Ych_i_* = *1*)) = *β*_0_ + *β*_1_*bfeeddur_t_* + *β*_2_*motherage_i_* + *β*_3_*childage_i_* + *β*_4_*sex_i_* + *β*_5_*married_i_* +
*β*_6_*educ_i_* + *β_7_employ_i_* + *β*_8_*antenatal_i_* + *β*_9_*caesarean_i_* + *β*_10_*birthsize_i_* + *β*_11_*HAZ_i_* + *β*_12_*WAZ_i_* +
*β*_13_*WHZ_i_* + *β*_14_*urban_i_* + *β*_15_*wealth_i_* + *β*_16_*net_i_* + *β*_17_*cookfuel_i_* + *β*_18_*geogreg_i_* (1)

*Ych_i_* is a dichotomous variable (0/1) that equals 1 if the child had a recent incident of ARI and 0 if otherwise. *bfeeddur_t_* is a vector for breastfeeding duration, with *t* capturing the different categories of breastfeeding. The duration of 1–6 months was used as the baseline category in the analysis. Covariates adjusted for include *motherage_i_*, *childage_i_*, *sex_i_, married_i_, educ_i_, and employ_i_,* which, respectively capture sociodemographic variables of mother’s age, child’s age, sex of the child, mother’s marital status, mother’s educational status, and mother’s employment status (in the past 12 months). *antenatal_i_*, *ceserean_i_*, and *birthsize_i,_* respectively capture maternal variables of the number of antenatal visits during pregnancy, mode of delivery (caesarean or natural birth), and birth size. *HAZ_i_, WAZ_i__,_* and *WHZ_i_* are vectors capturing the nutritional status of the child for different degrees of stunting, underweight, and wasting. The covariates *urban_i_, wealth_i_, net_i_, cookfuel_i_*, and *geogreg_i,_* respectively, capture the location of a household, wealth index of household, use of bed net by a child, type of cooking fuel used in a household, and geographic location of a household; to control for regional differences that exist across the various geographic areas in Nigeria, including economic conditions, cultural practices, access to services (e.g., healthcare, education), environmental factors, and policy differences. The categorization and coding of all the explanatory variables described above are provided in the Supplementary File (Table S1).

We conducted a sensitivity analysis to test the robustness of the main results of the model. A sample (n = 6762) of children whose mothers had stopped breastfeeding for at least 6 months before the survey was used in the statistical analysis. The result of this analysis is provided in Table S3. The R statistical package was used for all the analyses, and statistical significance was determined at the 95% confidence interval (*p* ≤ 0.05). The DHS survey involves a complex survey sampling procedure. Thus, DHS sampling weights were used in all our analyses to account for sampling and non-response errors. Lastly, the STROBE checklist was used in the writing of this manuscript [50]. See details of the STROBE in Table S2.

## 3. Results

### Descriptive Statistics

A total of 5391 children aged 24–59 months who had stopped breastfeeding for more than 12 months were included in the main analysis. About 17% of the children were in the age range of 24–35 months, while those aged 36–59 months comprised 83% of the sample. The mean maternal age in the sample was 31.6 (SD = 7.1) years, and male children made up about 52% of the sample. Ninety-two percent of the mothers in the sample did not have a higher education (post-secondary school education), and 26% were unemployed. About 55% of mothers in the sample had at least 4 antenatal visits when pregnant, and 99.8% had natural births. Almost 39% of children in the sample had an average birth size, and about 40% were either moderately or severely stunted. However, 77% and 82% of the children in the sample had normal z-scores for weight-for-age and weight-for-height. Other information on the household and geographical location of a household is shown in Table 1.

About 20 percent of children in the sample were breastfed for either 1–6 months or 7–12 months. Children breastfed for longer durations made up about 80% of the total sample, and approximately 3% percent of children had ARI (Table 2).

The Adjusted Odds Ratios (AOR) are shown in Table 3. Among all the breastfeeding categories analyzed, only the 19–24 month category was statistically significant at the 95% Confidence Interval (CI). The results indicate that compared to children breastfed for 1–6 months, children breastfed for 19–24 months duration had lower odds of ARI (AOR = 0.23, *p*-value = 0.04). Though we observed reduced incidents of the illness for the other durations, the estimates were not statistically significant at the 95% CI.

Most of the results for the sociodemographic, maternal, nutritional, and household variables were not statistically significant at the 95% CI. However, compared to children with a normal WAZ, severely underweight children had higher odds of recent incidents of ARI. Additionally, compared to the Northeastern part of Nigeria, all other regions except the Southeast were significantly associated with lower odds of recent incidents of ARI. 

The result of the sensitivity analysis is provided in Table S3. The estimates of the main explanatory variables suggest that there are long-term benefits of longer durations of breastfeeding. Compared to children breastfed for 1–6 months, the odds of recent incidents of ARI in children breastfed for longer durations are significantly lower.

## 4. Discussion

While an abundance of evidence exists regarding infectious outcomes in children as they relate to the short-term benefits of breastfeeding, there is limited evidence related to similar impacts after breastfeeding has stopped. More specifically, little is known about the long-term effects of breastfeeding durations on acute health outcomes after infancy. This study examined the association between “any breastfeeding” duration and recent incidents of ARI in young children after they had stopped breastfeeding. The results show that compared to children breastfed for 1–6 months, children breastfed for 19–24 months had lower odds of recent ARI post-infancy. We also observed reduced incidents of the illness for the other durations, but they were not statistically significant at the 95% CI. Though we did not find statistically significant relationships in most of the sociodemographic, maternal, nutritional, and household variables, compared to children with normal WAZ, we observed higher odds of recent incidents of ARI in severely underweight children.

The literature on the efficacy of breastfeeding in reducing childhood morbidity suggests a dose relationship between breastfeeding and reduced incidents of common childhood infections. A recent study by Vassilopoulou [51] provides more evidence on the efficacy of breastfeeding in reducing ARIs and emphasizes its promotion in vulnerable communities. Two other recent studies using DHS data from low and middle-income countries indicate that infants who were exclusively breastfed had lower odds of ARI and diarrhea [52,53]. 

We contribute to the literature on the dose relationship between breastfeeding and acute health outcomes in under-five children by examining the long-term impact of breastfeeding and utilizing data from a country and region where there is relatively less research on the topic. The findings from this study are consistent with previous studies that demonstrate the efficacy of longer durations of breastfeeding in reducing morbidity in infants [13,21,24,38,54]. Similar to this study, a study by Li et al. [55] demonstrated that children breastfed for more than 9 months had lower odds of ear, throat, and sinus infections at 6 years old. Other cross-sectional and cohort studies also show the positive effects of longer breastfeeding durations in reducing respiratory tract illnesses in infants and young children [20,56,57]. 

An interesting finding from this study is that it is consistent with the WHO recommendation that breastfeeding should continue as a supplement in children for up to 2 years and beyond [54,58]. Another interesting and novel finding from the results is the variation in the odds of ARI among children across the different geographical regions in Nigeria. The results suggest that, compared to the Northeastern region of the country, other regions have lower odds of the illness. This finding may likely result from the socioeconomic, ethnic, and religious differences across the regions. Future studies should further explore the regional differences in child health outcomes and their interactions with IYCF practices.

For many decades, programs and policies have been implemented globally to promote optimal IYCF practices [59]. The importance of early initiation of breastfeeding for infant exposure to colostrum in the first three days of life as well as exclusive breastfeeding has been emphasized, especially in low-resource settings, to protect infants from infections and promote growth [60,61]. In line with the global breastfeeding recommendations, the WHO and UNICEF jointly developed the Global Strategy for IYCF in 2002. The strategy provides a framework for action in some essential policy areas aimed at improving through optimal feeding, infant and young child growth, health, and overall survival [59]. Despite significant improvements in IYCF and child health outcomes since then, Nigeria still accounts for one of the highest under-five deaths globally, and ARI is a significant contributor to this burden [15]. This study provides further support for longer breastfeeding duration. Based on these findings, we recommend the strengthening of programs to encourage “any breastfeeding” duration for up to 19–24 months, as this can potentially reduce the incidents of respiratory illnesses even after breastfeeding has stopped. 

This study has some unique strengths. The NDHS is a large survey and is the best available health data from developing countries where comprehensive health data is otherwise absent. In addition, this study controlled for several potential confounding factors, including some not considered in past research on breastfeeding. Potential confounders such as regional effects have not been considered in many past studies, especially those conducted in sub-Saharan Africa. Another strength of the study is the study design, which reduces a type of reverse causality and temporal ambiguity common in many observational studies like this [62]. The study examined the relationship between durations of breastfeeding and ARI after breastfeeding has stopped. Thus, it can be argued that the children in the study were not breastfed due to the illness (reverse causality). In other words, the direction of effect is not bidirectional in the context of breastfeeding.

This study also has its limitations. The main limitation is the use of observational data that cannot fully account for differences in socio-demographic, physiological, and behavioral factors between study participants [24]. Though we attempted to adjust for potential confounding variables in the statistical analysis, differences among study participants likely remain and would impact the findings. It is also important to acknowledge that this study did not differentiate between exclusive and non-exclusive breastfeeding. The use of “any breastfeeding” duration may potentially reduce the true effect of breastfeeding and bias the results toward the null hypothesis [24,63]. Also, as stated above, children who were exclusively colostrum-fed in the first days after birth could have been exposed to more antibodies that may have a long-term impact in early childhood. This is also a potential confounder we did not account for in our model. Lastly, we acknowledge that the dataset used in this study is not very recent—about 15 years old. Our objective was to examine the long-term benefits of breastfeeding for longer durations in Nigeria, and the 2008 NDHS was the most recent survey with the variables to measure this effect. After 2008 and for surveys based on the DHS-VI, DHS-VII, or DHS VIII, information on the duration of breastfeeding and acute health outcomes is only collected for the youngest children or those who were still breastfeeding at the time of the survey [33]. Future studies should explore the use of alternative and more recent data sources. 

## 5. Conclusions

This study provides additional support for a dose relationship between breastfeeding and child health outcomes. Post-infancy and after breastfeeding has stopped, children who received breastmilk for 19–24 months had lower odds of recent episodes of ARIs. Nigeria is one of the countries contributing to the high child morbidity and mortality rates in Sub-Saharan Africa. Thus, findings from this study also have broader potential health, economic, and social policy implications not just for Nigeria but also for this region of the world.

Encouraging supplemental breastfeeding for a further 18 months after 6 months of exclusive breastfeeding, as recommended by the WHO, could significantly reduce the incidents of ARIs and, concurrently, under-five mortality rates in sub-Saharan Africa. Given that breastfeeding is highly accessible and a low-cost preventative public health intervention, this recommendation is a worthwhile endeavor.

## Figures and Tables

**Table 1:** Sociodemographic, maternal, nutritional, and household characteristics, 2008 NDHS.

Variables (N = 5391)	Frequency (Mean%)
**Child’s age (months)**	
24 to <36	903 (16.8)
36 to <48	2179 (40.4)
48 to 59	2309 (42.8)
**Mother’s age (years)**	31.57 (SD = 7.1)
**Child’s sex**	
Male	2774 (51.5)
Female	2617 (48.5)
**Mother’s education**	
No education	2231 (41.4)
Incomplete primary education	354 (6.6)
Complete primary education	922 (17.1)
Incomplete secondary education	617 (11.4)
Complete secondary education	837 (15.5)
Higher education	430 (8.0)
**Mother’s employment status (in the past 12 months)**	
Unemployed	1393 (25.8)
Employed	3989 (74.1)
**Antenatal visits during pregnancy**	
Less than 4 visits	1339 (24.8)
More than 4 visits	1646 (55.1)
**Mode of delivery**	
Natural birth	5265 (99.8)
Caeserean	122 (0.2)
**Birth size**	
Very small	182 (3.4)
Smaller than average	448 (8.4)
Average	2048 (38.5)
Larger than average	1593 (29.9)
Very large	1048 (19.7)
**Height-for-Age-Z-score (HAZ)**	
Normal (z-score is above minus 2 (−2.0) SD)	2696 (59.9)
Severely stunted (z-score is below minus 3 (−3.0) SD)	986 (21.9)
Moderately stunted (z-score is below −2.0 SD and above −3.0 SD)	816 (18.1)
**Weight-for-Age-Z-score (WAZ)**	
Normal (z-score is above minus 2 (−2.0) SD and less than +2 SD)	3690 (77.2)
Severely underweight (z-score is below minus 3 (−3.0) SD)	340 (7.6)
Moderately underweight (z-score is below −2.0 SD and above −3.0 SD)	636 (14.1)
Overweight (z-score is above +2 SD)	50 (1.1)
**Weight-for-Height-Z-score (WHZ)**	
Normal (z-score is above minus 2 (−2.0) SD and less than +2 SD)	3690 (82.0)
Severely wasted (z-score is below minus 3 (−3.0) SD)	372 (8.3)
Moderately wasted (z-score is below −2.0 SD and above −3.0 SD)	264 (5.9)
Overweight (z-score is above +2 SD)	172 (3.8)
**Household’s place of residence**	
Urban	3642 (67.6)
Rural	1749 (32.4)
**Wealth index of household**	
Poorest	1091 (20.2)
Poorer	1100 (20.4)
Middle	1058 (19.6)
Richer	1037 (19.2)
Richest	1105 (20.5)
**Use of bed net by child**	
No	4761 (88.3)
Yes	630 (11.7)
**Type of cooking fuel used in household**	
Solid fuels	4308 (72.4)
Clean fuels	1048 (19.6)
**Geographic location of household**	
North-central	1025 (19.0)
North-east	951 (17.6)
North-west	1233 (22.9)
South-east	568 (10.5)
South-south	769 (14.3)
South-west	845 (15.7)

“Any breastfeeding” duration and recent incidents of acute health outcomes in children.

**Table 2:** Breastfeeding durations and acute respiratory illness in children ages 24–59 months, 2008 NDHS.

Variables (n = 5391)	Frequency (%)
**Any breastfeeding**	
1–6 months	140 (2.6)
7–12 months	961 (17.8)
13–18 months	2269 (42.1)
19–24 months	1928 (35.7)
>24 months	93 (1.7)
**Acute Respiratory Illness in the last two weeks**	
Yes	154 (2.9)
No	5209 (97.1)

Breastfeeding duration and recent incidents of Acute Respiratory Illness in Children aged 24 to 59 months.

**Table 3:** Effect of any breastfeeding duration on recent incidents of acute respiratory infection among children aged 24–59 months in Nigeria (Adjusted Odds Ratios with CIs).

Variables	Acute Respiratory Illness
	(Adjusted Odds Ratios)
**Any breastfeeding (baseline = 1–6 months)**	
7–12 months	0.24 ^†^ [0.05–1.05]
13–18 months	0.32 ^†^ [0.08–1.18]
19–24 months	0.23 * [0.06–0.96]
>24 months	0.14 ^†^ [0.01–1.37]
Covariates	
Child’s age	1.00 [0.97–1.03]
Mother’s age	0.99 [0.67–1.02]
**Child’s Sex (baseline= male)**	
Male	1.25 [0.78–1.99]
**Mother’s Marital status (Baseline= unmarried)**	
Married	1.86 [0.72–4.84]
**Mother’s Education (baseline= no education)**	
Incomplete primary education	1.86 [0.89–3.91]
Complete primary education	0.96 [0.46–2.02]
Incomplete secondary education	1.03 [0.40–2.71]
Complete secondary education	0.78 [0.29–2.08]
Higher education	1.03 [0.12–1.94]
**Mother’s Employment status (Baseline= unemployed)**	
Employed	1.45 [0.78–2.67]
**Antenatal visits (baseline= less than 4 visits)**	
≥−4 visits	1.33 [0.74–2.40]
**Mode of child delivery (baseline = natural birth)**	
Ceserean	1.54 [0.32–7.43]
**Birth size of child (baseline= very small)**	
Smaller than Average	1.93 [0.45–8.29]
Average	0.92 [0.23–3.67]
Larger than Average	1.23 [0.30–4.97]
Very Large	1.34 [0.35–5.16]
**HAZ (baseline = normal)**	
Severely stunted	0.49 ^†^ [0.24–1.01]
Moderately stunted	0.66 [0.34–1.30]
**WAZ (baseline = normal)**	
Severely underweight	3.01 * [1.22–7.44]
Moderately Underweight	1.30 [0.59–2.87]
Overweight	1.11 [0.11–10.94]
**WHZ (baseline = normal)**	
Severely wasted	0.44 [0.15–1.32]
Moderately wasted	0.58 [0.12–2.79]
Overweight	0.75 [ 0.28–2.02]
**Household’s place of residence (baseline = rural)**	
Urban	0.55 [0.27–1.12
**Wealth index of household (baseline = poorest)**	
Poorer	0.74 [0.34–1.63]
Middle	1.03 [0.45–2.34]
Richer	0.90 [0.34–2.38]
Richest	0.78 [0.19–3.22]
**Use of bed net by child (baseline = no net)**	
Use of Net	0.54 [0.22–1.29]
**Type of cooking fuel used in the household (baseline = solid fuels)**	
Clean fuel	1.39 [0.45–4.26]
**Geographic location of household (baseline = North-east)**	
North-west	0.29 ** [0.14–0.62]
North-central	0.32 ** [0.16–0.66]
South-west	0.05 *** [0.01–0.21]
South-east	0.41 ^†^ [0.15–1.06]
South-south	0.44 * [0.20–0.95]

*** significant at *p*-value less than 0.001; ** significant at *p*-value less than 0.01; * significant at *p*-value less than 0.05. ^†^ Significant at a *p*-value less than 0.10; Confidence Intervals in parentheses.

## Data Availability

This study uses a publicly available secondary dataset, specifically the 2008 Nigerian DHS dataset. This dataset is publicly available through the DHS Program via https://www.dhsprogram.com/data/dataset_admin/login_main.cfm (accessed on: 8 August 2024).

## Supplementary Materials

The following supporting information can be downloaded at: https://www.mdpi.com/article/10.3390/children11091144/s1, Table S1: Operationalization and Coding of Exposure Variables and Covariates. Table S2: STROBE 2007 (v4) Statement—Checklist of items that should be included in reports of cross-sectional studies. Table S3: Effect of any breastfeeding duration on the acute health status of children, 2008 NDHS (Sample of children whose mothers had stopped breastfeeding at least 6 months before the survey, n = 6765).
