# Supplementary material for: Are There Benefits to Breastfeeding for Long Durations That Continue after Breastfeeding Has Stopped? An Analysis of Acute Respiratory Illness in Nigerian Children
Source: Children (Basel). 2024 Sep 21;11(9):1144. doi: 10.3390/children11091144 (PMC11430560; doi:10.3390/children11091144)
Supplement: Supplementary file 1 [file children-11-01144-s001.zip › children-3180244-supplementary.pdf]

Are There Long-term Benefits to Breastfeeding for Long Durations? An Analysis of Acute Respiratory Illness in Nigerian Children

Ademu LO et al.

Table S1: Operationalization and Coding of Exposure Variables and Covariates

**Variables**	**Category**
Breastfeeding durations	1. 0-6 months (Reference)<br>2. 7-12 months<br>3. 13-18 months<br>4. 18-24 month<br>5. >24 months
Sex of child	1. Female (Reference)<br>2. Male
Mother's education	0. No education (Reference)<br>1. Incomplete primary education<br>2. Complete primary education<br>3. Incomplete Secondary<br>4. Complete secondary<br>5. Higher education
Mother's employment status (in the past 12 months)	0. Unemployed (Reference)<br>1. Employed
Mother's marital status	0. Unmarried (Reference)<br>1. Married
Place of residence	0. Rural (Reference)<br>1. Urban
Wealth index of household	0. Poorest (Reference)<br>1. Poorer<br>2. Middle<br>3. Richer<br>4. Richest
Use of bed net by child	0. No (Reference)<br>1. Yes
Type of cooking fuel used in household	0. Solid fuel (Reference)<br>1. Clean fuel
Geographical location of residence	1. North-central (Reference)<br>2. North-east<br>3. North-west<br>4. South-east<br>5. South-south<br>6. South-west

Are There Long-term Benefits to Breastfeeding for Long Durations? An Analysis of Acute Respiratory Illness in Nigerian Children

Ademu LO et al.

Table S2: Reporting checklist for cross-sectional study. Based on the STROBE cross-sectional guidelines.(https://www.goodreports.org/reporting-checklists/strobe-cross-sectional/info/)

		Reporting Item	Page Number
**Title and abstract**			
Title	#1a	Indicate the study's design with a commonly used term in the title or the abstract	2
Abstract	#1b	Provide in the abstract an informative and balanced summary of what was done and what was found	2
**Introduction**			
Background/rationale	#2	Explain the scientific background and rationale for the investigation being reported	3-4
Objectives	#3	State the specific objectives, including any prespecified hypotheses	4
**Methods**			
Study design	#4	Present key elements of study design early in the paper	4-5
Setting	#5	Describe the setting, locations, and relevant dates, including periods of recruitment, exposure, follow-up, and data collection	4
Eligibility criteria	#6a	Give the eligibility criteria and the sources and methods of selection of participants.	5
	#7	Clearly define all outcomes, exposures, predictors, potential confounders, and effect modifiers. Give diagnostic criteria, if applicable	5-7
Data sources/measurement	#8	For each variable of interest give sources of data and details of methods of assessment (measurement). Describe the comparability of assessment methods if there is more than one group. Give information separately for exposed and unexposed groups if applicable.	4-5
Bias	#9	Describe any efforts to address potential sources of bias	6
Study size	#10	Explain how the study size was arrived at	5
Quantitative variables	#11	Explain how quantitative variables were handled in the analyses. If applicable, describe which groupings were chosen, and why	5-7
Statistical methods	#12a	Describe all statistical methods, including those used to control for confounding	6-7
Statistical methods	#12b	Describe any methods used to examine subgroups and interactions	6-7
Statistical methods	#12c	Explain how missing data were addressed	5
Statistical methods	#12d	If applicable, describe analytical methods taking account of the sampling strategy	NA
Statistical methods	#12e	Describe any sensitivity analyses	10-11
**Results**			
Participants	#13a	Report numbers of individuals at each stage of study—e.g. numbers potentially eligible, examined for eligibility, confirmed eligible, included in the study, completing follow-up, and analyzed. Give information separately for exposed and unexposed groups if applicable.	NA
Participants	#13b	Give reasons for non-participation at each stage	NA
Participants	#13c	Consider the use of a flow diagram	5

Descriptive data	#14a	Give characteristics of study participants (e.g. demographic, clinical, social) and information on exposures and potential confounders. Give information separately for exposed and unexposed groups if applicable.	8
Descriptive data	#14b	Indicate the number of participants with missing data for each variable of interest	NA
Outcome data	#15	Report numbers of outcome events or summary measures. Give information separately for exposed and unexposed groups if applicable.	9
Main results	#16a	Give unadjusted estimates and, if applicable, confounder-adjusted estimates and their precision (e.g., 95% confidence interval). Make clear which confounders were adjusted for and why they were included	9-10
Main results	#16b	Report category boundaries when continuous variables were categorized	9-10
Main results	#16c	If relevant, consider translating estimates of relative risk into absolute risk for a meaningful period	NA
Other analyses	#17	Report other analyses done—e.g., analyses of subgroups and interactions, and sensitivity analyses	NA
**Discussion**			
Key results	#18	Summarise key results with reference to study objectives	9-10
Limitations	#19	Discuss the limitations of the study, taking into account sources of potential bias or imprecision. Discuss both the direction and magnitude of any potential bias.	13
Interpretation	#20	Give a cautious overall interpretation considering objectives, limitations, multiplicity of analyses, results from similar studies, and other relevant evidence.	12-13
Generalisability	#21	Discuss the generalisability (external validity) of the study results	13
**Other Information**			
Funding	#22	Give the source of funding and the role of the funders for the present study and, if applicable, for the original study on which the present article is based	14

The STROBE checklist is distributed under the terms of the Creative Commons Attribution License CC-BY.
This checklist was completed on 19. June 2024 using https://www.goodreports.org/, a tool made by the https://www.equator-network.org/ in collaboration with https://www.penelope.ai/

Are There Long-term Benefits to Breastfeeding for Long Durations? An Analysis of Acute Respiratory Illness in Nigerian Children
Ademu LO et al.

Table S3: Effect of any breastfeeding duration on the acute health status of children, 2008 NDHS (Sample of children whose mothers had stopped breastfeeding at least 6 months before the survey, n=6,765)

Variables	Acute Respiratory Illness (Adjusted Odds Ratios)
**Any breastfeeding (baseline = 1-6months)**	
7 - 12 months	0.36† [0.12 - 1.04]
13 - 18 months	0.38† [0.14 - 1.06]
19 - 24 months	0.33* [0.15-0.79]
>24 months	0.28† [0.05 -1.51]
Married	0.90 [0.50-1.63]
Mother's age	0.99 [0.97-1.01]
Child's age	0.99 [0.97-1.01]
**Sex (Baseline= male)**	
Male	1.06 [0.76-1.45]
**Education (baseline= no education)**	
Incomplete primary education	1.81* [1.04 - 3.16]
Complete primary education	0.83 [0.45 - 1.55]
Incomplete secondary education	0.97 [0.46 - 2.03]
Complete secondary education	0.78 [0.37 - 1.66]
Higher education	0.69 [0.27 - 1.81]
**Place of residence (baseline= rural)**	
Urban	1.04 [0.62-1.75]
**Employment status (Baseline= unemployed)**	
Employed	1.31 [0.83-1.73]
**Use of mosquito net by child (baseline=no net)**	
Use of Net	0.62 [0.33-1.15]
**Source of cooking fuel (baseline=solid fuels)**	
Clean fuel	1.00 [0.46-2.14]
**Wealth index (baseline= poorest)**	
Poorer	0.95 [0.87-1.98]

Middle	1.26<br>[0.69-2.26]
Richer	0.94<br>[0.45-1.98]
Richest	0.85<br>[0.34- 2.17]
**Geopolitical region (baseline=North-east)**	
North-west	0.46***<br>[0.27-0.74]
North-central	0.29***<br>[0.17-0.51]
South-west	0.07***<br>[0.03-0.23]
South-east	0.58†<br>[0.31-1.09]
South-south	0.50*<br>[0.27-0.93]

**** significant at p-value less than 0.001; ** significant at p-value less than 0.01; * significant at p-value less than 0.05; † significant at p-value less than 0.10*
